# Fenofibrate enhances lipid deposition via modulating PPARγ, SREBP-1c, and gut microbiota in *ob/ob* mice fed a high-fat diet

**DOI:** 10.3389/fnut.2022.971581

**Published:** 2022-09-12

**Authors:** Ying Zhang, Xiu-Bin Jia, Yun-Chao Liu, Wen-Qian Yu, Yan-Hong Si, Shou-Dong Guo

**Affiliations:** ^1^College of Pharmacy and Pharmaceutical Sciences, Shandong First Medical University & Shandong Academy of Medical Sciences, Jinan, China; ^2^Innovative Drug Research Centre, School of Pharmacy, Institute of Lipid Metabolism and Atherosclerosis, Weifang Medical University, Weifang, China; ^3^College of Basic Medicine, Shandong First Medical University & Shandong Academy of Medical Sciences, Jinan, China

**Keywords:** obesity, NAFLD, triglyceride, PPARγ agonist, fibrates

## Abstract

Obesity is characterized by lipid accumulation in distinct organs. Presently, fenofibrate is a commonly used triglyceride-lowering drug. This study is designed to investigate whether long-term fenofibrate intervention can attenuate lipid accumulation in *ob/ob* mouse, a typical model of obesity. Our data demonstrated that fenofibrate intervention significantly decreased plasma triglyceride level by 21.0%, increased liver index and hepatic triglyceride content by 31.7 and 52.1%, respectively, and elevated adipose index by 44.6% compared to the vehicle group. As a PPARα agonist, fenofibrate intervention significantly increased the expression of PPARα protein in the liver by 46.3% and enhanced the expression of LDLR protein by 3.7-fold. However, fenofibrate dramatically increased the expression of PPARγ and SREBP-1c proteins by ~2.1- and 0.9-fold in the liver, respectively. Fenofibrate showed no effects on the expression of genes-related to fatty acid β-oxidation. Of note, it significantly increased the gene expression of *FAS* and *SCD-1*. Furthermore, fenofibrate modulated the gut microbiota. Collectively, long-term fenofibrate induces lipid accumulation in liver and adipose tissues in *ob/ob* mice by enhancing the expression of adipogenesis-related proteins and gut microbiota. These data suggest that fenofibrate may have limited effects on attenuating lipid deposition in obese patients.

## Introduction

The number of people with obesity is growing rapidly worldwide. According to the World Health Organization (WHO) report in 2021, the prevalence of obesity increased to 18% in 2016, and over 4 million people died as a result of being overweight or obese in 2017 (1). The rate of obesity in adults (>30 years old) is ~30% worldwide (2). Furthermore, the rate of increase of obesity in developing countries has been more than 30% higher than that of developed countries (1). It is worth noting that obesity is the starting event of many non-communicable diseases including non-alcoholic fatty liver disease (NAFLD), diabetes, hypertension, and cardiovascular disease (CVD) (3–5). Mechanistically, the onset and development of obesity is closely associated with a chronic positive energy balance modulated by multiple organs including brain, liver, pancreas, small intestine, muscle, and adipose tissues (6).

Lipid, such as triglyceride (TG), accumulation is a characteristic of obesity (7). Tissue-specific accumulation of TG is associated with distinct diseases. For instance, TG accumulation in the liver is defined as fatty liver diseases including NALFD and non-alcoholic steatohepatitis, which currently have no approved drugs for therapy (8, 9). Furthermore, TG is a residual risk factor of CVD (10). Theoretically, drugs with TG-lowering activity have a potential application for treatment of TG accumulation. Peroxisome proliferator activated receptor (PPAR) is a key transcription factor involved in obesity. Of note, PPARγ is a master regulator of adipogenesis, especially in humans (5, 11). On the contrary, activation of PPARα and β is found to reduce obesity and improve dyslipidemia (12, 13). Fenofibrate, a clinically used PPARα agonist, is found to reduce adipose tissue weight and size, plasma TG, and improve insulin sensitivity in high fat diet-induced obese C57BL/6J mice (14, 15). Mechanistically, fenofibrate can enhance the expression of the enzymes involved in fatty acid (FA) β-oxidation including acyl-CoA oxidase (ACOX) and liver X receptor in C57BL/6J mice and humans (14, 16, 17). However, fenofibrate shows limited beneficial effects on therapy of CVD (10).

*Ob/ob* mouse is leptin-deficiency due to *ob* gene depletion. This mouse model develops severe obesity as well as NAFLD and has been widely applied for investigation of obesity-associated diseases (18). PPARα-deficient *ob/ob* mice develop more severe obesity and hepatic steatosis compared to *ob/ob* mice (17). In a short-term (13-days intervention) study, fenofibrate prevents rosiglitazone-induced body weight gain and improves lipid profiles in *ob/ob* mice at the dosage of 100 mg/kg/d (19). However, fenofibrate is found to significantly increase the liver weight of the *ob/ob* mice after 2-weeks intervention at the dosage of 300 mg/kg/d (20). In a 12-weeks intervention, fenofibrate (50 mg/kg/d) elevates plasma level of TG as well as liver steatosis in *ob/ob* and low-density lipoprotein (LDL) receptor (LDLR)-double deficient mouse, a model of obesity and hyperlipidemia (21). It seems that fenofibrate with high dosage and/or long-term intervention can induce lipid accumulation in *ob/ob* mice. This study is designed to verify the above hypothesis and try to explain the underlying mechanisms of action of a long-term fenofibrate intervention.

## Materials and methods

### Materials

A bicinchoninic acid (BCA) protein quantitative kit (PC0020), and a hepatic TG assay kit (BC0625), fenofibrate (IF0040-500 mg, purity ≥ 99%), and phosphate buffered saline (PBS) powder were bought from Beijing Solarbio Science & Technology Co., Ltd. (Beijing, China). Assay kits for hepatic cholesterol and free fatty acid (FFA) were the products of Shanghai Lengton Bioscience Co., Ltd. (Shanghai, China). Assay kits for plasma total cholesterol (TC), high density lipoprotein (HDL) cholesterol (HDL-C), and TG were bought from Biosino Bio-technology and Science Incorporation (Beijing, China). Pellets of complete protease inhibitor were the product of Roche (Schweiz, Germany). Deionized water was obtained from a Milli-Q gradient system (Bedford, MA). The remaining reagents used in this study were of the analytical grade.

### Design of the experiment

Several previous studies suggest that the TG-lowering drug fenofibrate has inconsistent effects on obesity due to differences in intervention time and fenofibrate dosage. As patients with obesity need a long-term intervention, it is important to clarify whether long-term fenofibrate treatment benefits obesity using the well-acknowledged *ob/ob* mouse model. In this study, *ob/ob* mice were fed with a typical Western diet (as described later) for more than 3 months. Considering the potential side effects and the long-term intervention (22), the dosage of fenofibrate was determined as 20 mg/kg/d rather than 100 or 300 mg/kg/d that are used in short-term interventions (19, 20). At the end of this experiment, the plasma lipid profiles, body weight, liver weight, and epididymal fat weight were used to evaluate the effect of fenofibrate on lipid deposition in combination with other analysis methods. To investigate the underlying mechanisms, real-time quantitative polymerase chain reaction (RT-qPCR) and Western Blotting experiments were used for determining the expression of lipid metabolism-related genes and proteins, respectively. As accumulating evidence have demonstrated that gut microbiota may modulate obesity (10, 23), the effect of fenofibrate on gut microbiota in *ob/ob* mice was also analyzed in the present study.

### Animal grouping and intervention

This study was approved by the laboratory animals′ ethical committee of Shandong First Medical University (W202204150222). *Ob/ob* mice (male, 42–56 days old) were bought from Beijing HFK Bioscience Co., Ltd. (Beijing, China; License number: SCXK 2019-0008). High-fat chow was the product of Research Diets Inc. (Product #D12492, New Brunswick, NJ, USA). This chow was composed of casein (30 mesh, 200 g), L-cystine (3 g), maltodextrin 10 (125 g), sucrose (68.8 g), cellulose (BW200, 50 g), soybean oil (25 g), lard (245 g, containing 0.72 mg/g of cholesterol on average), mineral mix (S10026, 10 g), dicalcium phosphate (13 g), calcium carbonate (5.5 g), potassium citrate∙1 H_2_O (16.5), vitamin mix V10001 (10 g), choline bitartrate (2 g), and FD & C blue dye #1 (0.05 g). In this chow diet, protein, carbohydrate, and fat provide 20, 20, and 60% of the total calorie, respectively. After 7 days′ adaptive feeding, mice were randomly divided into two groups (eight mice in each group): the vehicle group (Vehicle, water by gavage) and the fenofibrate group (Fenofibrate, 20 mg/kg/d by gavage). Of note, fenofibrate is insoluble in water. Sodium carboxy methyl cellulose solution is generally used for preparation of suspension liquid when drugs are water-insoluble. However, carboxy methyl cellulose may affect the body weight and lipid profiles of the treated animals at the concentration of 2% (24). To eliminate the potential interference of the solvent medium, fenofibrate was made into suspension liquid (prepared every 2 days) via vortex with water before gavage. The average food intake and body weight were recorded weekly.

After 13-weeks intervention, the mice were anesthetized with 0.5% pentobarbital sodium by intraperitoneal injection (0.3 mL per mouse) after 12 h fasting. Blood was collected from the orbital sinus using heparinized capillary tubes under anesthesia. To remove the residual blood, the animals were perfused with ~10 mL of sterilized PBS through left ventricle before tissue sampling (25). To prevent potential contamination, the ileum and colon contents containing gut microbiota were carefully sampled in a horizontal flow clean bench. These ileum and colon contents were immediately frozen in liquid nitrogen and mailed to the Applied Protein Technology company with the protection of dry ice (Shanghai, China). Liver and epididymal fat were weighted, cut into pieces, and kept at −80°C before use. Liver or epididymal fat index was calculated according to the following formula: organ index = [(organ weight × 100%) ÷ body weight].

### Plasma analysis

Freshly sampled blood was centrifugated at 3,000 × *g* for 15 min at 4°C to obtain plasma. The levels of TC, TG, and HDL-C in the plasma were determined by the corresponding assay kits according to the manufacturers′ instructions. The absorbance of the samples was recorded on a Tecan Infinite 200 type multifunctional microplate reader (Molecular Devices, Lagerhausstrasse, Austria). Non-HDL-C was calculated by TC minus HDL-C. Additionally, 100 μL of mixed plasma in each group was separated by a Superose^TM^ 6 10/300 gel chromatography column that was linked to an ÄKTA fast protein liquid chromatography (FPLC) system. The column was eluted with mobile phase (0.9% NaCl) at a flow rate of 0.3 mL/min. The eluent was collected with an automatic collector with 0.5 mL in each fraction. In this study, 28 fractions were collected and the TC and TG levels in the very low-density lipoprotein (VLDL) and LDL fractions were determined by the commercially available assay kits.

### Liver homogenate analysis

Approximately 100 mg of liver was homogenized in 9-fold (w/v) freshly prepared PBS using a tissue grinder. The supernatant obtained after centrifugation at 1,100 × g for 10 min at 4°C was diluted at different ratio using PBS and then used to determine the concentration of TC, TG, and FFA according to the manufacturers' instructions. The protein content in each sample was determined by the BCA assay kit.

### Haematoxylin/Eosin (H & E) staining

The H & E experiment was carried out according to a previous publication (26). Liver samples were fixed in 4.0% paraformaldehyde at 4°C for 24 h and then embedded in paraffin. Approximately 5 micro-thick sections were cut with a sliding microtome (Leica, Germany). The slices were deparaffinized with xylene and gradient ethanol solutions, and then rinsed in distilled water for a few seconds. The nuclei were stained with heamatoxylin for 60 s, rinsed in distilled water, differentiated with acid alcohol (0.3%) for a few seconds, and then rinsed in distilled water for a few seconds. In the following, the slices were stained with 0.5% eosin for 60 s, dehydrated, cleared and mounted. The stained slices were visualized using an Olympus IX51 type microscope (Tokyo, Japan), and the images were recorded with a JVC 3-CCD camera at a magnification time of 200 × . In this study, the lipid droplets with a diameter > 10 μm were used for calculation of the average lipid droplet size. Furthermore, the number of the lipid droplets with a diameter > 20 μm was counted within a randomly chosen area of 20,000 μm^2^ in each slice for calculation of the average lipid droplet number.

### RT-qPCR

Total ribonucleic acid (RNA) was isolated from the liver tissue using TRIzol reagent (CW0580s, Beijing, China) according to the manufacturer's instruction. The concentration and purity of total RNAs were measured by a nanodrop ultraviolet spectrophotometer. The total RNAs were considered to be pure if the ratio of A260/A80 > 1.90. A HiFiScript complementary deoxyribonucleic acid (cDNA) synthesis kit (CW2569M, Beijing, China) was used to prepare cDNA using 1.0 μg of total RNA that obtained from each sample. RT-qPCR was carried out in an ABI QuantStudio 3 PCR system (Thermo Fisher, USA) using an UltraSYBR mixture (low ROX) (CW2601, Beijing, China). This method was used to detect the expression of lipid metabolism-related genes including sterol regulatory element binding protein (*SREBP*)*-1c, PPAR*γ, *PPAR*α, carnitine palmitoyltransferase (*CPT*)-*1*α, *CPT-2, ACOX-1*, stearoyl Coenzyme A desaturase-1 (*SCD-1*), and fatty acid synthase (*FAS*) as listed in Table 1. The specific primers were synthesized by QingDao Ribo Xingke Biotechnology Co., Ltd. (Qingdao, China). The program was set as following: initial denaturation at 95°C for 10 min followed by 40 cycles of 95°C (denaturation temperature) for 10 s, 60°C (annealing temperature) for 30 s, and 72°C (elongating temperature) for 32 s. The relative expression of target gene was calculated by the method of 2^−*DDCt*^ based on the housekeeping gene glyceraldehyde-3-phosphate dehydrogenase (*GAPDH*).

**Table 1:** The primers used for the polymerase chain reaction (PCR) reaction.

**Primer**		**Sequences (5^′^-3^′^)**
GAPDH	Forward	TGCCACCCAGAAGACTGTG
	Reverse	ATGTAGGCCATGAGGTCCAC
PPARγ	Forward	GGTTGACACAGAGATGCCAT
	Reverse	GCTGGAGAAATCAACTGTGG
CPT-1α	Forward	GACCGCGCTCTTAGGACTAC
	Reverse	GAAAGCCCACTCTTCTGCCT
CPT-2	Forward	TCCTCCTACTCAGGACGCAA
	Reverse	TTGGGATCTTCCGCTCACAC
ACOX1	Forward	CGCCACCTTCAATCCAGAGT
	Reverse	TCTGCGATGCCAAATTCCCT
PPARa	Forward	GCAGCTCGTACAGGTCATCA
	Reverse	TACCTACGCTCAGCCCTCTT
SREBP-1c	Forward	TGGACGAGCTGGCCTTCGGT
	Reverse	GGCCAGCGGCAGGCTAGATG
SCD-1	Forward	CATCATTCTCATGGTCCTGCT
	Reverse	CCCAGTCGTACACGTCATTTT
FAS	Forward	CATCCACTCAGGTTCAGGTG
	Reverse	AGGTATGCTCGCTTCTCTGC

### Western blotting

Total proteins of the mice liver were extracted using radioimmunoprecipitation assay buffer containing complete protease inhibitor. Equal amounts of protein (~40 μg) were subjected to sodium dodecyl sulfate-polyacrylamide gel electrophoresis (SDS-PAGE) and then transferred onto polyvinylidene fluoride membranes (Millipore, ROPB61783, USA) by electroblotting (27). The membranes were first blotted with 5% of defatted milk power for 2 h at room temperature. Then, the membranes were blotted with the corresponding primary and secondary antibodies listed as follows: a mouse monoclonal antibody against SREBP-1c (2A4) (SANTA CRUZ, USA, sc-13551, 1:500), a rabbit polyclonal antibody against PPARγ (Proteintech, USA, 16643-1-AP, 1:1000), a mouse monoclonal antibody against PPARα (Abcam, USA, ab97609, 1:800), a mouse monoclonal antibody against β-actin (Abcam, USA, ab8226, 1:1000), a rabbit polyclonal antibody against LDLR (Abcam, USA, ab30532, 1:200), and secondary antibodies including a goat anti-rabbit IgG H&L (HRP) (Abcam, USA, ab6721, 1:3000) and a goat anti-mouse IgG H&L (HRP) (Abcam, USA, ab205719, 1:5000). After blotting, immunoblots were revealed by enhanced chemiluminescence solution from Millipore (WBKLS0500, USA). The images were recorded on a Clinx ChemiScope 6000 pro system (Shanghai, China). The expression of the target proteins was normalized by housekeeping protein β-actin.

### Detection of plasma apolipoprotein (Apo)

As for the plasma, each 10 μL plasma sample was added with 90 μL of PBS and 20 μL 5 × loading buffer. The mixture was mixed well and heated at 80°C for 10 min. Approximately 5 (for Apo A-I) or 10 μL (for Apo B) plasma medium was subjected to 12.5% (for Apo A-I) or 6% (Apo B) SDS-PAGE. Finally, the membranes were botted with the corresponding primary and secondary antibodies listed as follows: a rabbit polyclonal antibody against albumin (Proteintech, 16475-1-AP, Wuhan, China, 1:10,000), a mouse monoclonal antibody against Apo A-I (Proteintech, 66206-1-Ig, Wuhan, China, 1:1000), and a rabbit polyclonal antibody against Apo B (Proteintech, 20578-1-AP, Wuhan, China, 1:800). The expression of the target proteins was normalized by albumin.

### Gut microbiota analysis

Total genomic deoxyribonucleic acid (DNA) was extracted from the fecal samples using CTAB/SDS method. The concentration and purity of DNA were determined by 1.0% agarose gel electrophoresis. The concentration of DNA was adjusted to 1.0 μg/mL using sterile water. The V3-V4 region of the 16S rRNA genes were amplified using the 341F-806R primers. PCR products were purified with an AxyPrepDNA gel extraction kit (AXYGEN). Sequencing library was generated using a NEB Next^®^ Ultra^TM^ DNA library Prep Kit for Illumina (NEB, USA) following the manufacturer's instructions. Finally, the library was sequenced on an Illumina Miseq/HiSeq2500 platform and 250/300 bp paired-end reads were generated. The paired-end reads were assigned to each sample based on the unique barcodes. Sequence analysis was carried out by the UPARSE software package using the UPARSE-OTU and UPARSE-OTUref algorithms.

### Data analysis

All the bioassay results were expressed as mean ± standard deviation (SD) for at least three independent experiments. Statistical analysis was performed by Student-*t*-test, and the differences were considered to be significant at a *p* < 0.05.

## Results

### Fenofibrate reduced plasma TG level in *ob/ob* mice

**Figure 1:**
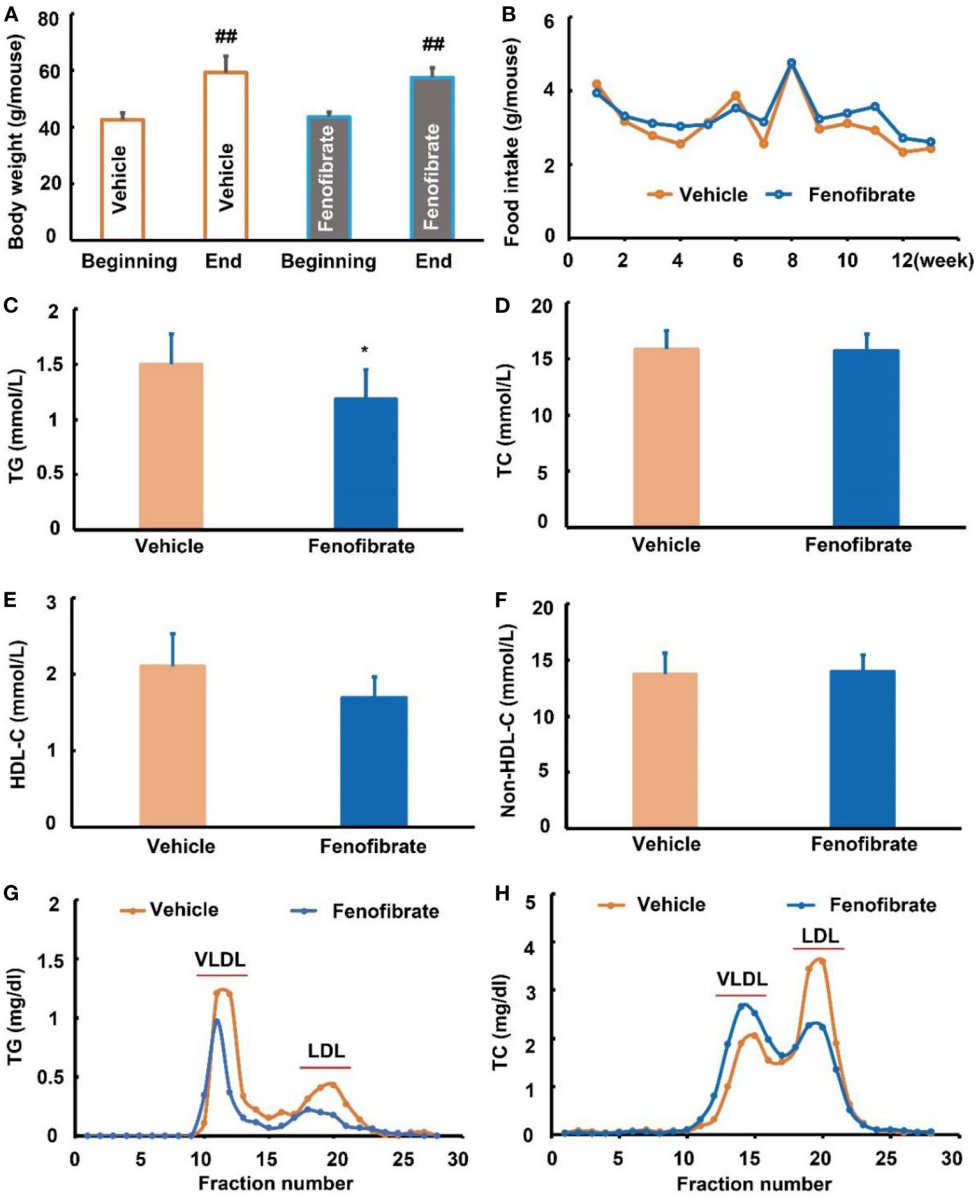
Effect of fenofibrate on the body weight, food intake, and lipid profiles of the *ob/ob* mice. **(A)** body weight of the mice at the beginning and end of the study; **(B)** food intake of mice in each group; **(C)** plasma triglyceride (TG) levels; **(D)** plasma total cholesterol (TC) levels; **(E)** plasma high density lipoprotein cholesterol (HDL-C) levels; **(F)** plasma non-HDL-C levels; **(G)** plasma TG profiles of very low-density lipoprotein (VLDL) and low-density lipoprotein (LDL) fractions after ÄKTA fast protein liquid chromatography (FPLC) separation; **(H)** plasma TC profiles of VLDL and LDL fractions after ÄKTA FPLC separation. Data were expressed as mean ± SD, and the statistical analysis was performed by Student-*t*-test. ^##^ means significantly different at *p* < 0.01 *vs*. the beginning body weight of the mice; * means significantly different at *p* < 0.05 *vs*. Vehicle group. Vehicle: mice were treated with 0.5 mL of water by gavage for 13 weeks (*n* = 7); Fenofibrate: mice were treated with 0.5 mL of fenofibrate suspension liquid at the dose of 20 mg/kg/d by gavage for 13 weeks (*n* = 8).

In this study, one *ob/ob* mouse in the vehicle group was removed from the final statistical analysis due to a great body weight loss induced by an unknown reason (Supplementary Data 1). As shown in Figure 1A, the beginning body weight of the mice in the vehicle and fenofibrate group had no significant difference. The high-fat diet induced ~40% body weight gain in mice. However, the final body weight of the mice in the vehicle and fenofibrate group showed no significant difference. Furthermore, fenofibrate intervention had no obvious influence on the food intake of the mice (Figure 1B). In the plasma, fenofibrate treatment dramatically decreased the TG level by ~21.0% compared to that of vehicle group (Figure 1C, *p* < 0.05). However, fenofibrate intervention had no significant effect on the levels of TC, HDL-C, and non-HDL-C (Figures 1D–F). The results of ÄKTA FPLC demonstrated that fenofibrate lowered the levels of TG in both VLDL and LDL fractions (Figure 1G) compared with the vehicle group, which were consistent with the reduced plasma TG level in the fenofibrate group. In this study, fenofibrate decreased the cholesterol level in the LDL fractions and increased the cholesterol level in the VLDL fractions (Figure 1H). This result was consistent with the non-significant difference of the plasma TC and non-HDL-C between vehicle and fenofibrate groups (Figures 1D,F). Additionally, fenofibrate showed no effect on the expression of Apo A-I (**Figure 3A**) and Apo B48 proteins (**Figure 3B**). However, fenofibrate intervention significantly reduced the expression of Apo B100 by ~107.5% (**Figure 3B**, *p* < 0.05).

### Fenofibrate enhanced lipid accumulation in the liver

**Figure 2:**
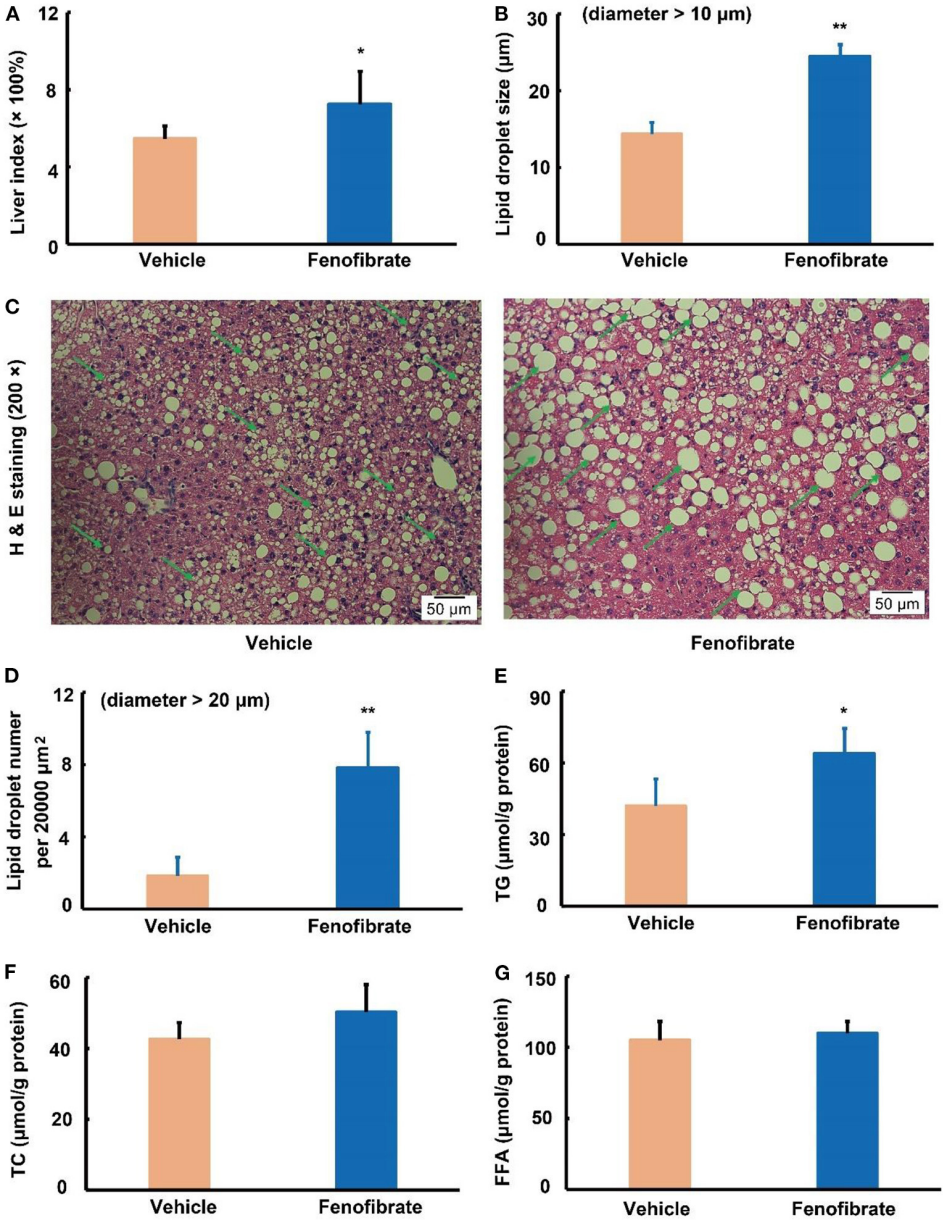
Effect of fenofibrate on the liver index, lipid droplet formation, and lipid contents in the liver of the *ob/ob* mice (*n* = 7 for the Vehicle group and *n* = 8 for the fenofibrate group unless otherwise specified). **(A)** liver index was calculated according to the following formula: liver index = [(liver weight × 100%) ÷ body weight]; **(B)** the average lipid droplet size, the lipid droplets with a diameter > 10 μm were used for this statistical analysis in five distinct areas; **(C)** the typical images of H and E staining (*n* = 5); **(D)** the average number of the lipid droplet with a diameter > 20 μm per 20,000 μm^2^; **(E)** triglyceride (TG) content of the liver; **(F)** total cholesterol (TC) content of the liver; **(G)** free fatty acid (FFA) content of the liver. Data were expressed as mean ± SD, and the statistical analysis was performed by Student-*t*-test. * means significantly different at *p* < 0.05 *vs*. Vehicle group; ** means significantly different at *p* < 0.01 *vs*. Vehicle group. Some typical lipid droplets were pointed out using green arrows. Vehicle: mice were treated with 0.5 mL of water by gavage for 13 weeks; Fenofibrate: mice were treated with 0.5 mL of fenofibrate suspension liquid at the dose of 20 mg/kg/d by gavage for 13 weeks.

As shown in Figure 2A, fenofibrate intervention obviously increased the liver index by ~31.7% compared to the vehicle group (*p* < 0.05). Of note, fenofibrate intervention significantly increased the lipid accumulation in the liver (Figure 2C). In this study, the lipid droplet with a diameter > 10 μm was analyzed for comparation of the lipid droplet size, and the results demonstrated that fenofibrate increased the lipid droplet size by 71.2% compared to the vehicle group (Figure 2B, *p* < 0.01). Furthermore, the average number of the lipid droplet with a diameter > 20 μm per 20,000 μm^2^ in fenofibrate group was ~3.3-fold of that in the vehicle group (Figure 2D, *p* < 0.01). In line with these findings, fenofibrate intervention increased TG content from 42.0 ± 11.3 μg/g protein to 63.9 ± 10.8 μg/g protein in the liver (Figure 2E, *p* < 0.05). However, the content of TC and FFA in the liver had no significant difference between the vehicle group and fenofibrate group (Figures 2F,G).

### Fenofibrate activated the expression of PPARα and adipogenesis-related proteins in the liver

**Figure 3:**
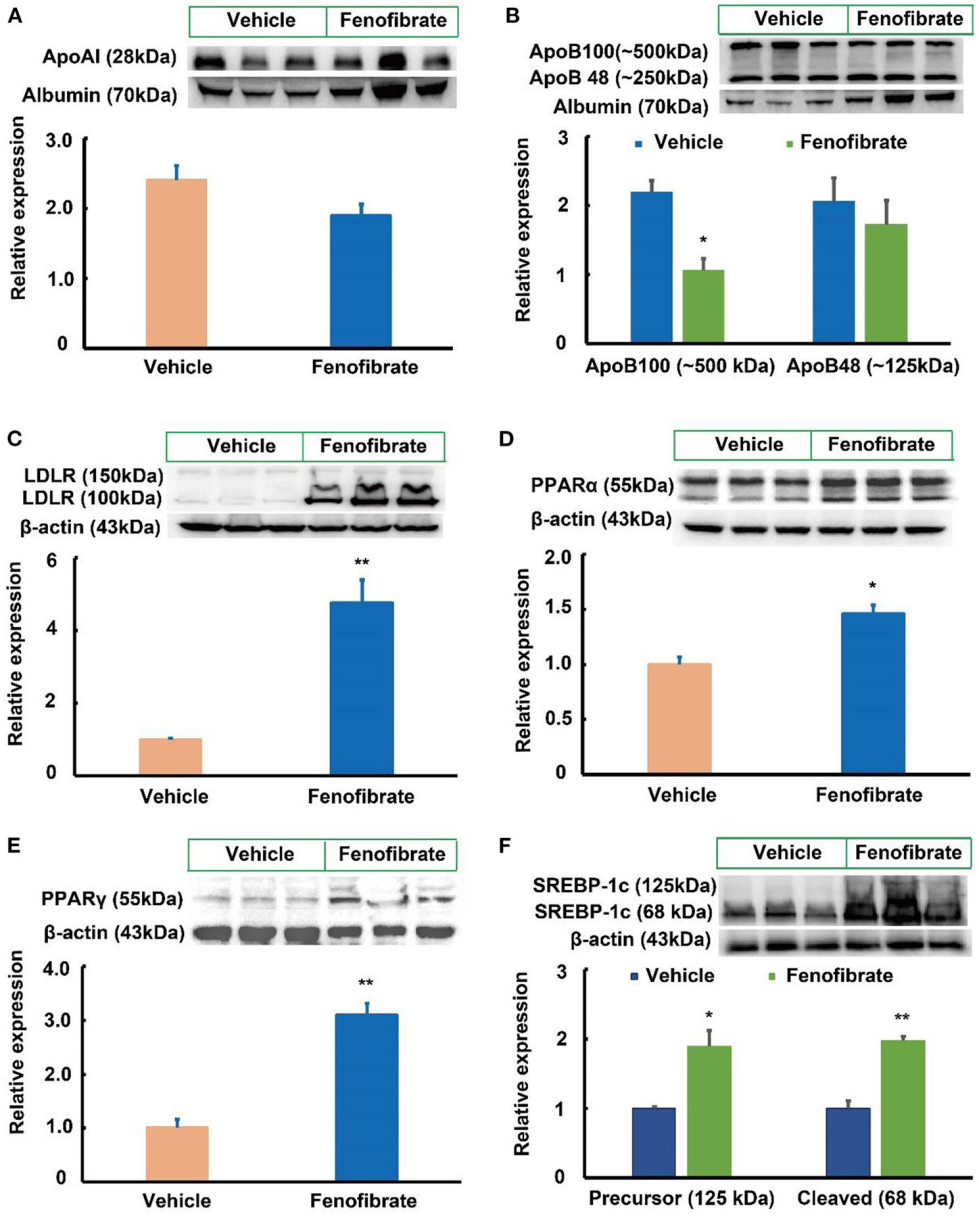
Effect of fenofibrate on the expression of proteins involved in lipid metabolism in the plasma and liver of the *ob/ob* mice (*n* = 6). **(A)** protein expression and densitometric quantification of plasma apolipoprotein (Apo) A-I; **(B)** protein expression and densitometric quantification of plasma Apo B; **(C)** protein expression and densitometric quantification of low-density lipoprotein receptor (LDLR) in the liver; **(D)** protein expression and densitometric quantification of peroxisome proliferator activated receptor (PPAR) α in the liver; **(E)** protein expression and densitometric quantification of PPARγ in the liver; **(F)** protein expression and densitometric quantification of sterol regulatory element binding protein (SREBP)-1c in the liver. Data were expressed as mean ± SD, and the statistical analysis was performed by Student-*t*-test. * means significantly different at *p* < 0.05 *vs*. Vehicle group; ** means significantly different at *p* < 0.01 *vs*. Vehicle group. Vehicle: mice were treated with 0.5 mL of water by gavage for 13 weeks; Fenofibrate: mice were treated with 0.5 mL of fenofibrate suspension liquid at the dose of 20 mg/kg/d by gavage for 13 weeks.

LDLR plays a key role in clearance of LDL and VLDL particles from the circulation (10). In the present study, fenofibrate significantly increased the expression of LDLR protein by ~3.7-fold compared to the vehicle group (Figure 3C, *p* < 0.01). As a PPARα agonist, fenofibrate intervention significantly increased the expression of PPARα protein by 46.3% compared with the vehicle group (Figure 3D, *p* < 0.05). Of note, fenofibrate treatment dramatically increased the expression of PPARγ protein by ~2.1-fold in the liver of the *ob/ob* mice compared to the vehicle group (Figure 3E, *p* < 0.01). Furthermore, fenofibrate enhanced the expression of the precursor and cleaved SREBP-1c by ~88.5% (*p* < 0.05) and 97.3% (*p* < 0.01), respectively (Figure 3F).

**Figure 4:**
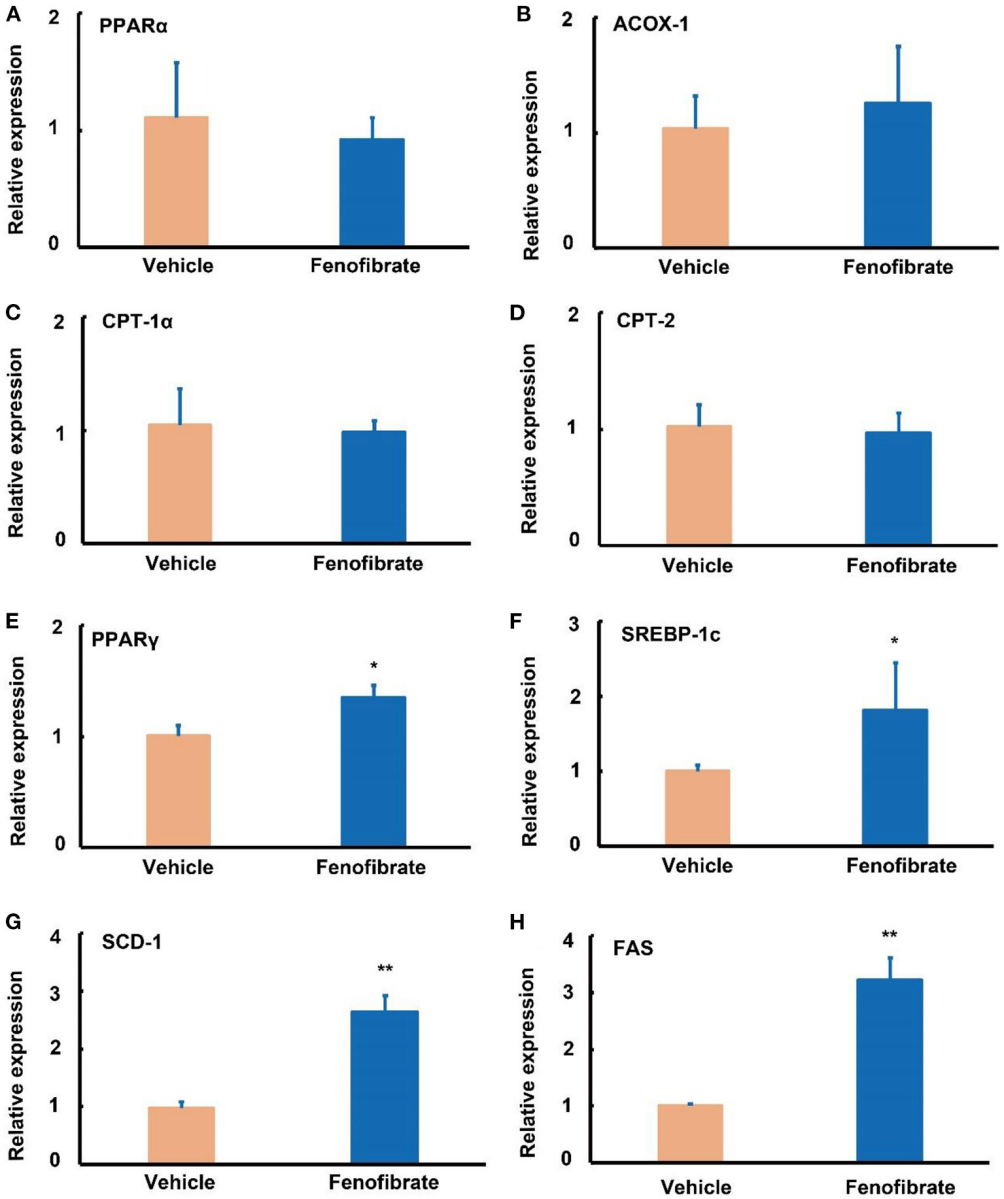
Effect of fenofibrate on the expression of genes involved in lipid metabolism in the liver of the *ob/ob* mice. Gene relative expression of **(A)** peroxisome proliferator activated receptor (PPAR) α (*n* = 12); **(B)** acyl-CoA oxidase (ACOX)-1 (*n* = 12); **(C)** carnitine palmitoyltransferase (CPT)-1α (*n* = 12); **(D)** CPT-2 (*n* = 12); **(E)** PPARγ (*n* = 7); **(F)** sterol regulatory element binding protein (SREBP)−1c (*n* = 9); **(G)** stearoyl Coenzyme A desaturase-1 (SCD-1) (*n* = 4); **(H)** fatty acid synthase (FAS) (*n* = 4). The RT-qPCR experiment was repeated for one more time to confirm the gene expression trends of PPARα, ACOX-1, CPT-1α, CPT-2, and SREBP-1c. Data were expressed as mean ± SD, and the statistical analysis was performed by Student-*t*-test. * means significantly different at *p* < 0.05 *vs*. Vehicle group; ** means significantly different at *p* < 0.01 *vs*. Vehicle group. Vehicle: mice were treated with 0.5 mL of water by gavage for 13 weeks; Fenofibrate: mice were treated with 0.5 mL of fenofibrate suspension liquid at the dose of 20 mg/kg/d by gavage for 13 weeks.

Compared with the vehicle group, fenofibrate had no significant effects on the gene expression of *PPAR*α, *ACOX-1, CPT*-1α, and *CPT-2* (Figures 4A–D). However, fenofibrate significantly increased the gene expression of *PPAR*γ by ~31% and elevated the gene expression of *SREBP-1c* by ~83% (Figures 4E,F, *p* < 0.05). Furthermore, fenofibrate dramatically enhanced the gene expression of *SCD-1* and *FAS* by 1.8- and 2.2-fold, respectively (Figures 4G,H, *p* < 0.05).

### Fenofibrate enhanced adipose index in the *ob/ob* mice

**Figure 5:**
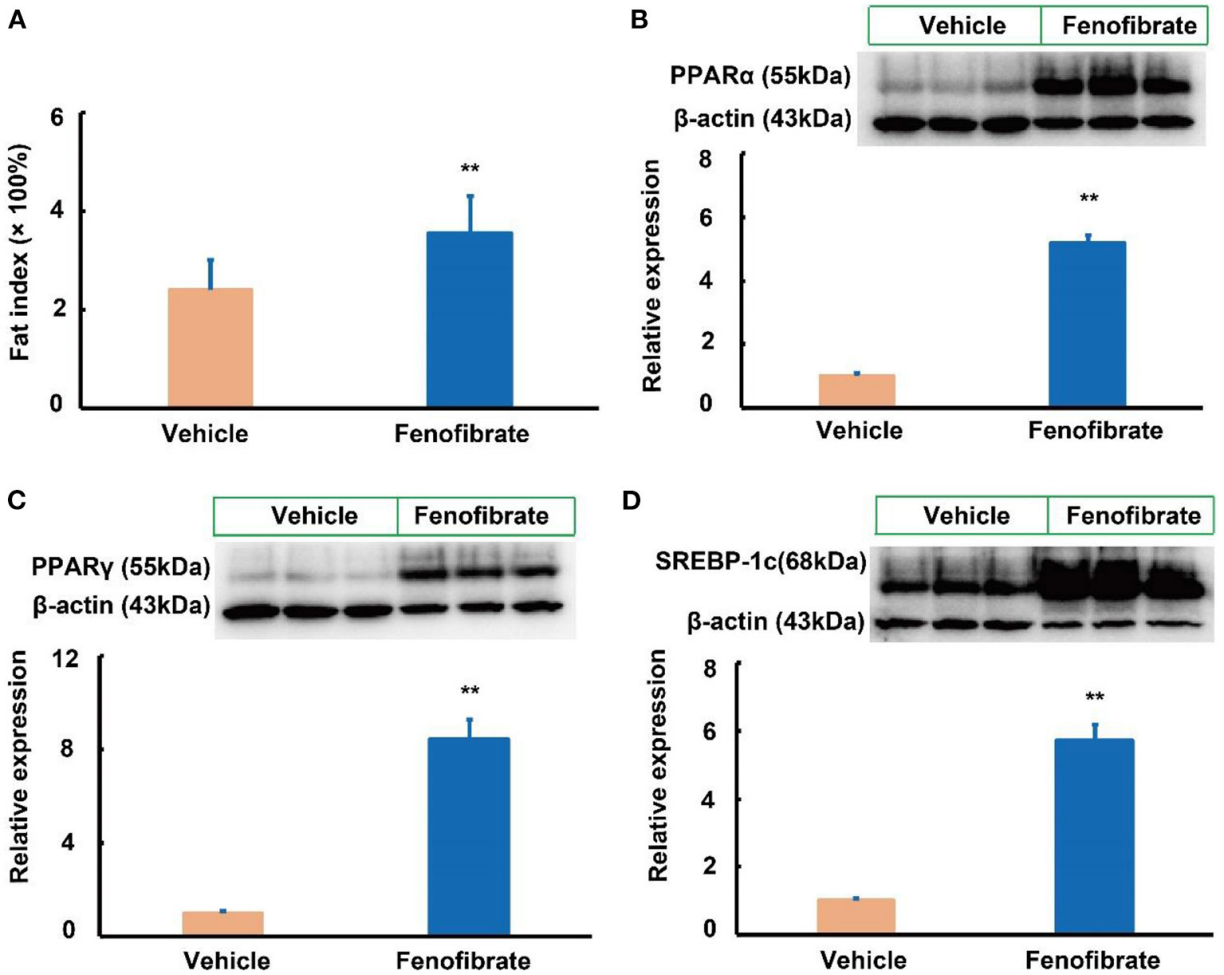
Effect of fenofibrate on the fat index and expression of proteins involved in lipid metabolism in the white adipose of the *ob/ob* mice (*n* = 6 unless otherwise specialized). **(A)** fat index was calculated according to the following formula: fat index = [(white adipose weight × 100%) ÷ body weight] (*n* = 7 for the Vehicle group and *n* = 8 for the Fenofibrate group). Protein expression and densitometric quantification **(B)** peroxisome proliferator activated receptor (PPAR) α; **(C)** PPARγ; **(D)** sterol regulatory element binding protein (SREBP)-1c. Data were expressed as mean ± SD, and the statistical analysis was performed by Student-*t*-test. ** means significantly different at *p* < 0.01 *vs*. Vehicle group. Vehicle: mice were treated with 0.5 mL of water by gavage for 13 weeks; Fenofibrate: mice were treated with 0.5 mL of fenofibrate suspension liquid at the dose of 20 mg/kg/d by gavage for 13 weeks.

In this study, fenofibrate treatment significantly elevated the fat index by 44.6% compared to the vehicle group (Figure 5A, *p* < 0.01). In the white adipose tissue, fenofibrate intervention significantly increased the expression of PPARα protein by 4.18-fold compared with the vehicle group (Figure 5B, *p* < 0.05). Of note, fenofibrate treatment dramatically enhanced the expression of PPARγ and SREBP-1c proteins by 7.45- and 4.71-fold, respectively (*p* < 0.01, Figures 5C,D).

### Fenofibrate modulated the gut microbiota

**Figure 6:**
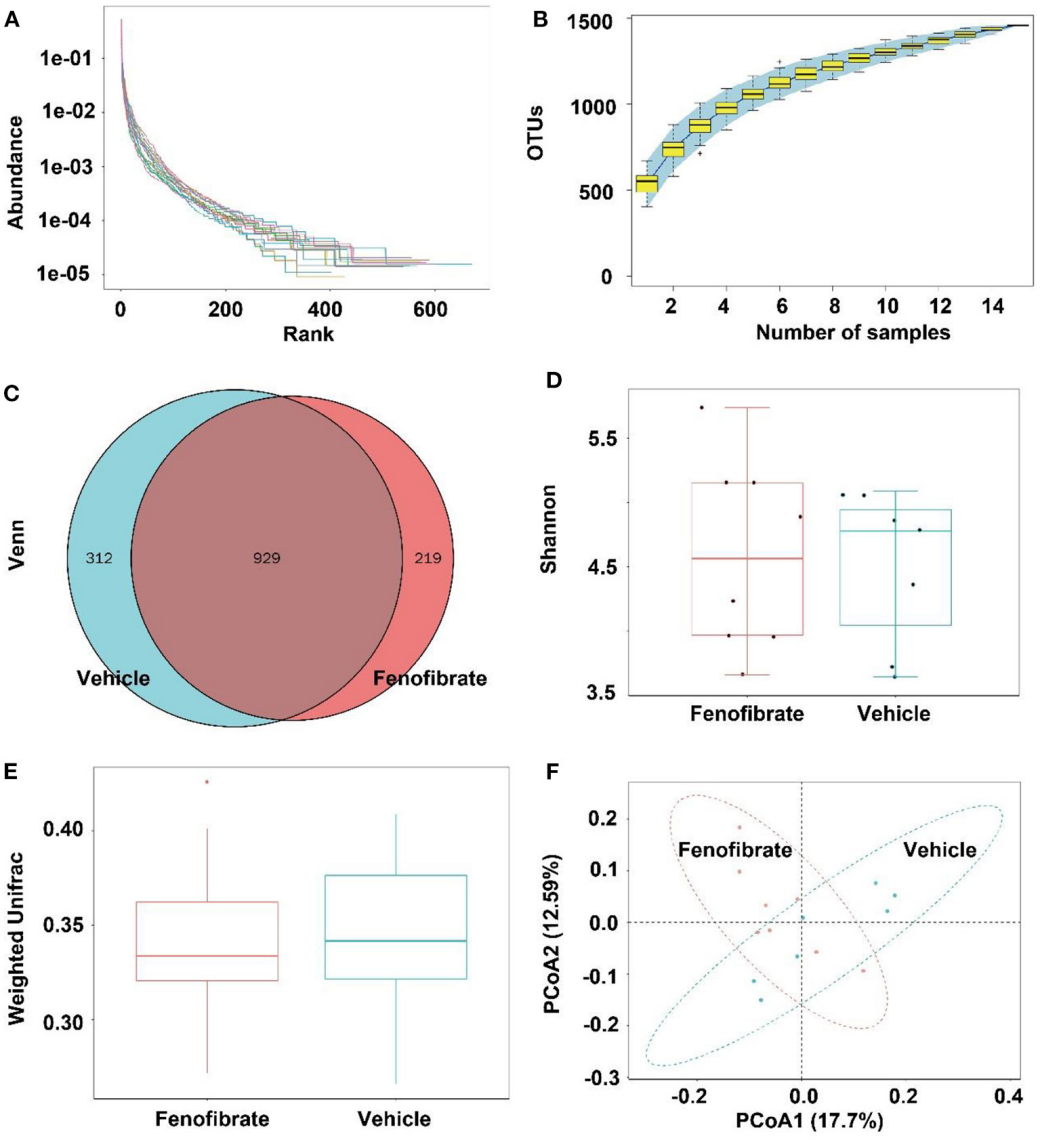
Effect of fenofibrate on the gut microbiota of the *ob/ob* mice. **(A)** rank-abundance curves; **(B)** species accumulation curves; **(C)** OTU Venn graph; **(D)** α diversity (Shannon graph); **(E)** β diversity (Weighted Unifrac); **(F)** principal co-ordinates analysis. Vehicle: mice were treated with 0.5 mL of water by gavage for 13 weeks (*n* = 7); Fenofibrate: mice were treated with 0.5 mL of fenofibrate suspension liquid at the dose of 20 mg/kg/d by gavage for 13 weeks (*n* = 8).

**Figure 7:**
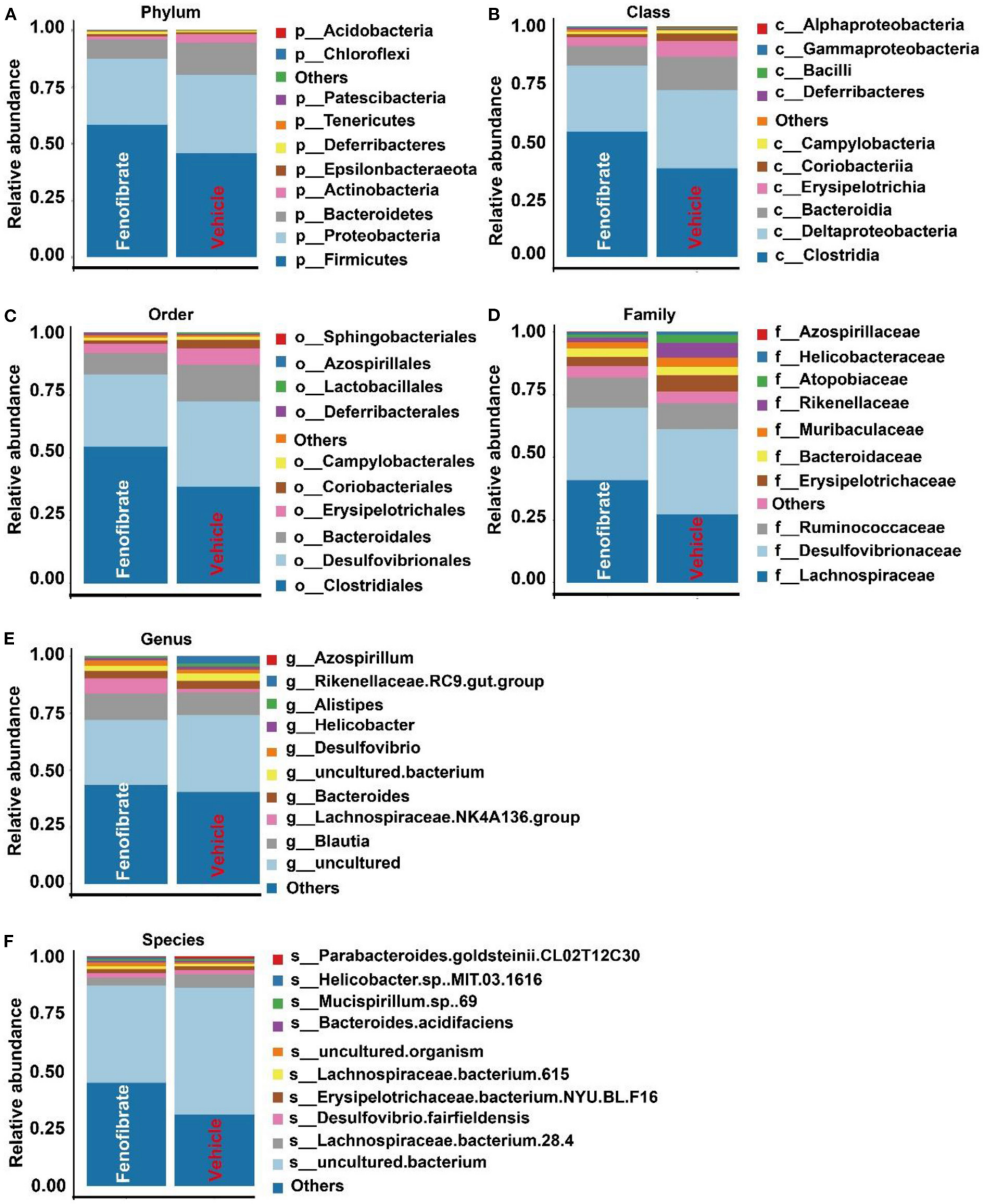
Effect of fenofibrate on the gut microbiota species in different classification levels. The top 10 microbiota species in phylum **(A)**, class **(B)**, order **(C)**, family **(D)**, genus **(E)**, and species **(F)** levels. Vehicle: *ob/ob* mice were treated with 0.5 mL of water by gavage for 13 weeks (*n* = 7); Fenofibrate: *ob/ob* mice were treated with 0.5 mL of fenofibrate suspension liquid at the dose of 20 mg/kg/d by gavage for 13 weeks (*n* = 8).

Gut microbiota is correlated with TC and TG metabolism (10, 23, 28). The rank-abundance curves (Figure 6A) and the species accumulation curves (Figure 6B) demonstrated that species richness of the gut microbiota was great enough in this study. Of note, a lower number of OTU was observed in fenofibrate intervention group compared to that of vehicle group (Figure 6C). The α-diversity (Figure 6D) and β-diversity analysis (Figure 6E) suggested that the species in the fenofibrate group have a greater dispersion and a lower value of median number. The principal co-ordinates analysis (PCoA) showed that the microbiota species have a mild difference between fenofibrate and vehicle group (Figure 6F). The top 10 microbiota species in different classification levels including phylum, class, order, family, genus, and species, were shown in Figure 7. Of note, fenofibrate intervention elevated the average relative abundance of *p_Firmicutes* (0.584 ± 0.145 vs. 0.457 ± 0.076) and reduced the *p_Bacteroidetes* (0.085 ± 0.095 vs. 0.144 ± 0.156) and *p_Actinobacteria* (0.015 ± 0.019 vs. 0.035 ± 0.067) at the phylum level; it increased the average relative abundance of *c_Clostridia* (0.544 ± 0.146 vs. 0.384 ± 0.082, *p* < 0.05) and decreased *c_Bacteroidia* (0.085 ± 0.095 vs. 0.144 ± 0.156) and *c_Erysipelotrichia* (0.037 ± 0.039 vs. 0.067 ± 0.008) at the class level; fenofibrate elevated the average relative abundance of *o_Clostridiales* (0.544 ± 0.146 vs. 0.384 ± 0.082, *p* < 0.05) and reduced *o_Bacteroidales* (0.085 ± 0.095 vs. 0.144 ± 0.156) and *o_Erysipelotrichales* (8.456 E-4 ± 2.182 E-4 vs. 4.852 E-4 ± 2.375 E-4, *p* < 0.05) at the order level; it increased the relative abundance of *f_Lachnospiraceae* (0.408 ± 0.150 vs. 0.271 ± 0.067) and *f_Ruminococcaceae* (0.122 ± 0.031 vs. 0.102 ± 0.047) at the family level. Furthermore, fenofibrate enhanced the abundance of *g_Lachnospiraceae.NK4A136.group* (0.068 ± 0.062 vs. 0.016 ± 0.009) and *g_[Eubacterium]* brachy group (6.08 E-4 ± 3.299 E-4 vs. 2.228 E-4 ± 1.337 E-4, *p* < 0.05) at the genus level and reduced the relative abundance of *s_uncultured.bacterium* (0.422 ± 0.132 vs. 0.553 ± 0.058, *p* < 0.05), *s_Lactobacillus crispatus* (5.719 E-6 ± 1.014 E-5 vs. 8.124 E-5 ± 6.429 E-5, *p* < 0.01)and *s_Lachnospiraceae.bacterium.28.4* (0.034 ± 0.052 vs. 0.057 ± 0.048) at the species level (Figure 7 and Supplementary Data 2).

**Figure 8:**
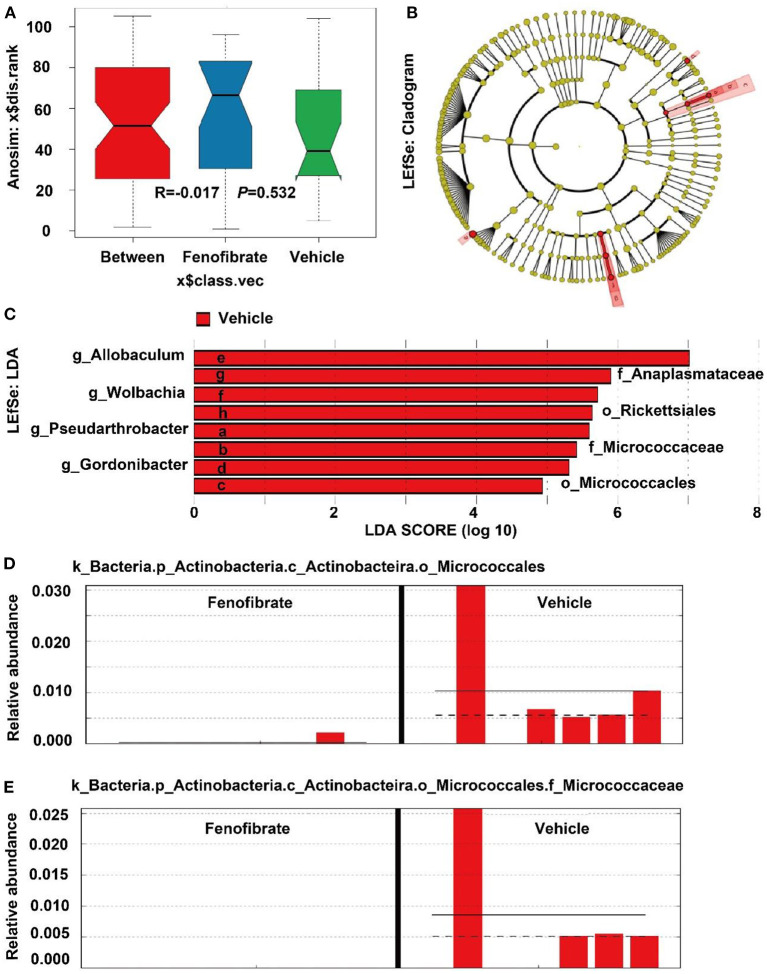
Anosim and LDA Effect Size (LEfSe) analysis of the gut microbiota species. **(A)** Anosim analysis; **(B)** LEfSe analysis shown by cladogram; **(C)** LEfSe analysis shown by LDA score; **(D,E)** the relative abundance of some microbiota biomarkers in different samples. Vehicle: *ob/ob* mice were treated with 0.5 mL of water by gavage for 13 weeks (*n* = 7); Fenofibrate: *ob/ob* mice were treated with 0.5 mL of fenofibrate suspension liquid at the dose of 20 mg/kg/d by gavage for 13 weeks (*n* = 8).

However, the Anosim analysis demonstrated that the R value was less than zero (*R* = −0.017) and the *P*-value was >0.05 (*P* = 0.532), suggesting the species difference within groups was greater than that between groups (Figure 8A). This may be explained by the great dispersion of the microbiota species in the fenofibrate group (Figure 6D). According to the Linear Discriminant Analysis (LDA) Effect Size (LEfSe) analysis, fenofibrate intervention showed a depletion of *Rickettsiales* and *Micrococcacles* at the order level, and displayed a depletion of *Anaplasmataceae* and *Micrococcaceae* at the family level. Furthermore, fenofibrate depleted the abundance of *Gordonibacter, Allobaculum, Wolbachia*, and *Pseudarthrobacter* at the genus level (Figures 8B,C). Further analysis demonstrated that the *k_Bacteria.p_Actinobacteria.c_Actinobacteira.o_Micrococcales, k_Bacteria.p_Actinobacteria.c_Coriobacteriia.o_Coriobacteriales, k_Bacteria.p_Firmicutes.c_Erysipelotrichia.o_Erysipelotrichales. f_Erysipelotrichaceae.g_Allobaculum*, and *k_Bacteria.p_Proteobacteria.c _Alphaproteobacteria.o_Richettsiales* could be assigned as biomarkers. For instance, fenofibrate significantly decreased the abundance of *k_Bacteria.p_Actinobacteria.c_Actinobacteira.o_Micrococcales* (Figure 8D) and especially the *Micrococcaceae* at the family level (Figure 8E).

## Discussion

The prevalence of obesity has greatly increased the onset of many non-communicable diseases (8, 9). Obesity is characterized by lipid accumulation (7). Fenofibrate is the most commonly used TG-lowering drug mainly by activation of PPARα. In this study, we demonstrated for the first time that long-term fenofibrate intervention significantly reduced the plasma level of TG and dramatically increased lipid accumulation in the liver and adipose tissues in *ob/ob* mice. Mechanistically, fenofibrate significantly enhanced the expression of LDLR, PPARα, PPARγ, and SREBP-1c proteins and modulated the gut microbiota.

Fenofibrate is a fibric acid derivative developed for therapy of patients with hypertriglyceridemia as well as other dyslipidemias via activation of PPARα (29). In this study, long-term (13 weeks) fenofibrate intervention significantly decreased the plasma level of TG accompanied with the activation of PPARα, which was consistent with previous findings in *ob/ob* mice that were treated with fenofibrate for ~2 weeks (19, 20). Furthermore, we demonstrated that fenofibrate could reduce the TG level in VLDL and LDL particles. In line with the TG alterations, fenofibrate intervention significantly decreased the expression of Apo B 100, but not Apo B48, which was consistent with previous publications (30–32). Mechanistically, fenofibrate reduces the production rate of hepatic Apo B100 and increases the fractional catabolic rate (31, 32). In this study, fenofibrate significantly enhanced the expression of LDLR protein in the liver, which is responsible for the clearance of Apo B-containing lipoproteins from circulation (10). In *ob/ob* and *LDLR*-double deficient mouse, fenofibrate (50 mg/kg/d) elevates plasma level of TG and mildly elevated TC level, suggesting the important role of LDLR for clearance of non-HDL particles in *ob/ob* mice (21). However, short-term fenofibrate (100 mg/kg, 13 d) may also reduce plasma TC in ob/ob mice (19). As *ob/ob* mice with a C57BL/6J background are non-HDL dominant, the TC and TG levels in the HDL fractions are hard to be detected after ÄKTA FPLC separation due to the more than 150 times of dilution of plasma during the separation. Therefore, we assayed the level of HDL-C using the freshly prepared plasma rather than the fractions obtained by ÄKTA FPLC separation. Unlike the findings in humans (33) and rats (34), fenofibrate could not increase the level of HDL-C in *ob/ob* mice, which was consistent with the unchanged plasma level of Apo A-I in this study. Moreover, this data was consistent with our previous study in Apo E-deficient mice, which also have a C57BL/6J background (26). Another study finds that fenofibrate can significantly reduce HDL-C by ~30% in C57Bl mice (35). In a recent comparative study, fenofibrate (0.1% w/w) reduces plasma levels of TG, TC, and LDL/VLDL-C, in male C57BL/6J mice fed a high-fat diet, but not in the mice fed a standard-fat diet (36). Furthermore, 21 weeks′ fenofibrate intervention (0.05% w/w) significantly decreases the plasma levels of TG and TC in female ovariectomized C57BL/6J mice (37). Another study indicates that fenofibrate (25 mg/kg) significantly reduces plasma TG and increases TC and LDL-C in high-fat diet induced C57BL/6 mice after 38 days intervention (38). These results suggest that fenofibrate may have different effects on modulating HDL-C, TC, and TG based on the animal species, feeding diets as well as intervention time. Furthermore, fenofibrate has better effect on TG-lowering as well as on activation of the downstream genes of PPARα in male than in female C57BL/6J mice, suggesting the effects of fenofibrate can be affected by endogenous molecules such as estrogen (39, 40).

Liver plays a key role in lipid metabolism and is a main organ for FA β-oxidation. In this study, fenofibrate significantly elevated the liver index in *ob/ob* mice. This result is consistent with several previous studies, which demonstrate that short-term (14 d) and long-term (12 weeks) fenofibrate intervention increases liver index in *ob/ob* and *ob/ob* and LDLR-double deficient mice, respectively (20, 21). In this study, we demonstrate for the first time that long-term fenofibrate intervention enhances liver steatosis in *ob/ob* mice, which is consistent with the findings in *ob/ob* and LDLR-double deficient mice (21). Another study indicates that fenofibrate has no effect on hepatic TG content and PPARγ agonist pioglitazone significantly increases hepatic TG content in C57BL/6J mice fed a high-fat diet (41). However, fenofibrate intervention significantly decreases hepatic TG in female ovariectomized or male C57BL/6J mice (37, 42). Fenofibrate as well as various endogenous ligands, such as long-chain FAs, can modulate FA β-oxidation at the transcriptional level via activation of PPARα, thereby promoting β-oxidation and reducing availability of FAs for TG synthesis (43, 44). Mechanistically, fenofibrate activates the transcriptional activity of PPARs through peroxisome proliferator response elements, thereby modulating the genes involved in lipid metabolism (44, 45). Unlike the observations in C57BL/6J mice (37, 38), fenofibrate intervention does not reduce the plasma level of FFA in this study. However, short-term fenofibrate intervention (~14 d) is found to reduce plasma level of FFA in *ob/ob* mice (19, 20). These data suggest that the intervention time may have affected the effect of fenofibrate on FFA. Moreover, fenofibrate had no effect on the gene expression of PPARα and its target gene ACOX-1 in this study, which is a rate-limiting enzyme of FA β-oxidation in mitochondria and peroxisome (43). In line with our data, 14 weeks′ fenofibrate intervention (0.05% w/w) successfully reduces the plasma levels of TG, however, fenofibrate shows no effect on the gene expression of PPARα and its heterodimerization partner retinoid X receptor in both male and female C57BL/6J mice fed a high-fat diet (39). CPTs mediate the activation and transport of FAs into mitochondria and are rate-limiting enzymes involved in FA β-oxidation. Of note, CPT-1α is the liver isoform of CPT and locates at the outer mitochondrial membrane. It is responsible for converting acyl-CoA into acyl-carnitines (46). CPT-2 locates at the inner side of the mitochondria and plays a key role in long-chain FA oxidation (47). In the present study, fenofibrate had no effect on the expression of FA oxidation related genes as mentioned above including ACOX-1, CPT-1α, and CPT-2. These data suggest that long-term fenofibrate intervention may have limited effects on FA β-oxidation in *ob/ob* mice. However, fenofibrate significantly increases the gene expression of ACOX, 3-hydroxyacyl-CoA dehydrogenase, which are the downstream genes of PPARα, in male C57BL/6J mice or female ovariectomized C57BL/6J mice fed a high-fat diet (37, 39, 48). Therefore, fenofibrate shows different effects on activation of PPARα downstream genes in *ob/ob* and C57BL/6J mice.

We tried to investigate why fenofibrate enhances lipid accumulation in the liver as well as in adipose tissue of the *ob/ob* mice. As a PPARα agonist, fenofibrate significantly increased the expression of PPARα protein, suggesting fenofibrate did work in *ob/ob* mice in this study. Upon obesity, such as in leptin-deficient (*ob/ob*) mice, PPARγ and its target gene Fsp27 are activated in the liver to promote FA storage as lipid droplets (49). In C57BL/6J mice fed a high-fat diet, long-term fenofibrate intervention (30 mg/kg, 20 weeks) can decrease the expression of Fsp27 by 51% accompanied with reductions in hepatic TG and epididymal fat without affecting serum TG (42). Fenofibrate also reduces the body weight and the adipose tissue weight in male C57BL/6J mice fed a high-fat diet, but not in the mice fed a standard-fat diet (36, 38). Furthermore, 21 weeks′ fenofibrate intervention (0.05% w/w) significantly decreases body weight, adipose tissue weight, hepatic TG, and the number of ballooned hepatocyte, in female ovariectomized C57BL/6J mice (37). Interestingly, fenofibrate only reduces the body weight and white adipose tissue weight in male C57BL/6J mice (39). These data suggest that *ob/ob* gene deletion as well as sex hormone may contribute to the different modulatory effects of fenofibrate. Although fenofibrate is a selective agonist of PPARα, this molecule is demonstrated to activate the expression of PPARγ in different models (26, 50). As is known, PPARγ promotes adipogenesis at the early stage and is highly expressed in patients with NAFLD (50, 51). *In vitro*, PPARγ plays key roles in adipocyte differentiation and maturation (27). *In vivo*, PPARγ activation (pioglitazone, ~15 mg/kg), but not PPARα activation (fenofibrate, ~150 mg/kg), induces multilocularization of adipocytes and increases subcutaneous, epididymal, and retroperitoneal fat accumulation in male C57BL/6, *db/db*, and *ob/ob* mice fed normal diet for 3 weeks (52). Therefore, the lipid accumulation promoting capacity of fenofibrate in *ob/ob* mice can be partially attributed to its effect on enhancing the expression of PPARγ. What's more, fenofibrate enhanced the SREBP-1c signaling pathway, which regulates FA and TG synthesis at the transcriptional level (53). FAS and SCD-1 are target genes of SREBP-1c and are key enzymes involved in *de novo* FA synthesis and the formation of mature adipocyte (50, 54). For instance, SCD-1 can convert stearoyl-CoA to oleoyl-CoA, which is an important intermediate product in TG synthesis (54, 55). Therefore, TG accumulation in liver and adipose tissues of the fenofibrate-treated *ob/ob* mice can be explained by the significantly enhanced levels of SREBP-1c, and its target genes including *FAS* and *SCD-1*. The gene expression is not always consistent with the protein expression. Therefore, we presume that fenofibrate may influence the gene expression of PPARα by modulating other unknow molecules, such as endogenous metabolites as well as small interfering RNA and long-chain non-coding RNA. The unchanged expression of PPARα is consistent with its unchanged downstream genes including *ACOX-1* and *CPT*. This hypothesis needs to be investigated in future. Of note, several studies have demonstrated that dual PPARα and PPARγ agonists are effective for treatment of metabolic diseases including NAFLD in *ob/ob* mice and rats (34, 56). However, fenofibrate significantly increased lipid accumulation in the liver and epididymal fat in this study. Therefore, the hepatic TG accumulation induced by fenofibrate may be mainly attributed to the enhanced expression of SREBP-1c signaling pathway in this study.

Furthermore, gut microorganisms are closely correlated with various diseases including obesity by interacting with distinct organs through their metabolites (23, 57). In line with a previous study, fenofibrate had limited influence on gut microbiota (58). *Bacteroidetes* (*Porpyromonas* and *Prevotella*) and *Firmicutes* (*Ruminococcus, Clostridium*, and *Eubacteria*) are the two dominant microbial phyla and take 9–42% and 30–52% of the gut microbiota, respectively (59). Accumulating evidence have demonstrated that reductions in the ratio of *Bacteroidetes*/*Firmicutes* promote the development of obesity in different models (10, 23, 60). In this study, fenofibrate reduced the abundance of *Bacteroidetes* and increased the abundance of *Firmicutes*. On the contrary, fenofibrate (0.1% w/w) is found to reverse the reduced ratio of *Bacteroidetes*/*Firmicutes* in C57BL/6J mice fed a high-fat diet (36). Furthermore, the reduction of *Actinobacteria* and increase of *Clostridium* (*Firmicutes*) are also associated with adipogenesis, and the increase of *Erysipelotrichaceae* and *Allobaculum* is correlated with reduction of weight gain (59, 61). Fenofibrate was found to reduce the abundance of *p_Actinobacteria, g_Allobaculum*, and *o_Erysipelotrichales* and increase *c_Clostridia* in *ob/ob* mice. These data suggest that fenofibrate enhances obesity via modulating gut microbiota. A previous study also demonstrates that fenofibrate can decrease the abundance of *Allobaculum* and *Erysipelotrichaceae* in choline-deficient diet-induced NAFLD (62).

The non-digestible carbohydrates or some amino acids are converted into short chain FAs (SCFAs), which have multiple physiological effects on host health including lipid homeostasis (23, 60). It is shown that *Ruminococcaceae, Lachnospiraceae*, and *Micrococcaceae* are positively correlated with obesity (63). Although fenofibrate treatment decreased the abundance of *Micrococcaceae*, it significantly increased the relative abundance of *Ruminococcaceae* and *Lachnospiraceae* at the family level. On the contrary, fenofibrate reduces the abundance of *Ruminococcaceae* and *Lachnospiraceae* in C57BL/6J mice fed a high-fat diet (36). The depletion of *Gordonibacter* at the genus level may also contribute to the adipogenesis effect of fenofibrate (64, 65). Furthermore, *Gordonibacter* identified as *Actinobacteria* species converts ellagic acid into urolithins, which exert an anti-inflammatory activity (28). Therefore, fenofibrate may elevate inflammation via modulation of gut microbiota in *ob/ob* mice. Of note, fenofibrate intervention promoted the butyrate-producing and propionate-producing *Ruminococcaceae* and *Lachnospiraceae* at the family level, and increased the abundance of the SCFA-producing *Lachnospiraceae.NK4A136.group* at the genus level (23, 66). Butyrate and propionate have been demonstrated to activate β-oxidation in the mitochondria by stimulating PPARγ (23, 67). However, fenofibrate reduced the relative abundance of *Bacteroidia, Actinobacteria, Allobaculum*, and *Anaplasmataceae*, which are also involved in SCFAs production (59, 68). A recent study demonstrates that fenofibrate enhances the production of SCFAs, including acetic acid, propionic acid, and butyric acid, more efficiently in male C57BL/6J mice fed a high-fat diet than those fed a standard-fat diet through modulation of the gut microbiota, thereby ameliorating inflammation (36). However, whether fenofibrate can promote the production of SCFAs in *ob/ob* mice need to be further investigated in the future because fenofibrate exhibits different modulatory effects on the gut microbiota in *ob/ob* mice compared to that in C57BL/6J mice (36).

## Conclusion

Although fenofibrate significantly reduces plasma level of TG, this molecule enhances lipid accumulation in the liver and adipose tissues in *ob/ob* mice. Mechanistically, fenofibrate improves plasma TG via enhancing the expression of LDLR protein. However, it shows limited effect on the genes involved in FA β-oxidation. Of note, this molecule significantly elevated the expression of PPARγ, SREPB-1c, FAS, and SCD-1, which are closely correlated with TG synthesis and adipocyte differentiation and maturation. Collectively, long-term fenofibrate intervention enhances lipid deposition in *ob/ob* mouse, a typical model of obesity. These data suggest that long-term fenofibrate intervention may have limited effects in obese patients with NAFLD. However, these differences may be induced by the *ob/ob* gene deletion, which needs to be further clarified in the future.

## Data availability statement

The data presented in the study are deposited in the figshare repository (10.6084/m9.figshare.20745994)/Supplementary material.

## Ethics statement

The animal study was reviewed and approved by Shandong First Medical University (W202204150222).

## Author contributions

YZ designed this study and provided funding. Y-HS, X-BJ, Y-CL, and W-QY carried out the experiments and performed the statistical analysis. Y-HS wrote the manuscript. S-DG contributed to the design, funding, and re-editing of the manuscript. All authors contributed to the article and approved the submitted version.

## Funding

This work was supported by National Natural Science Foundation of China (82070469, 81770463, 81600681), Key Research and Development Project of Shandong Province (2019GSF108260), and Academic Promotion Plan of Shandong First Medical University (2019QL013, 2019LJ003).

## Conflict of interest

The authors declare that the research was conducted in the absence of any commercial or financial relationships that could be construed as a potential conflict of interest.

## Publisher's note

All claims expressed in this article are solely those of the authors and do not necessarily represent those of their affiliated organizations, or those of the publisher, the editors and the reviewers. Any product that may be evaluated in this article, or claim that may be made by its manufacturer, is not guaranteed or endorsed by the publisher.

## Abbreviations

ACOX: acyl-CoA oxidase
Apo: apolipoprotein
BCA: bicinchoninic acid
cDNA: complementary deoxyribonucleic acid
CPT: carnitine palmitoyltransferase
CVD: cardiovascular disease
DNA: deoxyribonucleic acid
FA: fatty acid
FAS: fatty acid synthase
FFA: free fatty acid
FPLC: fast protein liquid chromatography
GAPDH: glyceraldehyde-3-phosphate dehydrogenase
HDL: high density lipoprotein
HDL-C: high density lipoprotein cholesterol
H & E: haematoxylin/eosin
LDA: linear discriminant analysis
LEfSe: LDA Effect Size
LDL: low-density lipoprotein
LDLR: low-density lipoprotein receptor
NAFLD: non-alcoholic fatty liver disease
PCoA: principal co-ordinates analysis
PCR: polymerase chain reaction
PPAR: peroxisome proliferator-activated receptor
RNA: ribonucleic acid
RT-qPCR: real-time quantitative PCR
SCD-1: stearoyl coenzyme A desaturase-1
SCFA: short chain fatty acid
SDS-PAGE: sodium dodecyl sulfate-polyacrylamide gel electrophoresis
SREBP: sterol regulatory element-binding protein
TC: total cholesterol
TG: triglyceride
VLDL: very low-density lipoprotein.
