# Supplementary material for: Fenofibrate enhances lipid deposition via modulating PPARγ, SREBP-1c, and gut microbiota in ob/ob mice fed a high-fat diet
Source: Front Nutr. 2022 Sep 12;9:971581. doi: 10.3389/fnut.2022.971581 (PMC9511108; doi:10.3389/fnut.2022.971581)
Supplement: Supplementary file 1 [file Data_Sheet_1.ZIP › WB Raw data.pdf]

Western blotting (Plasma)

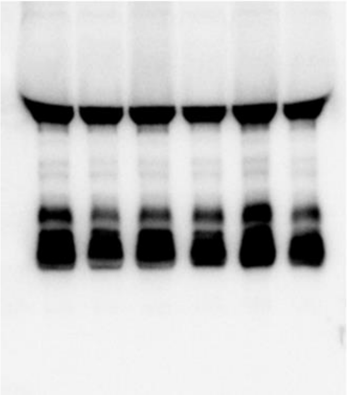

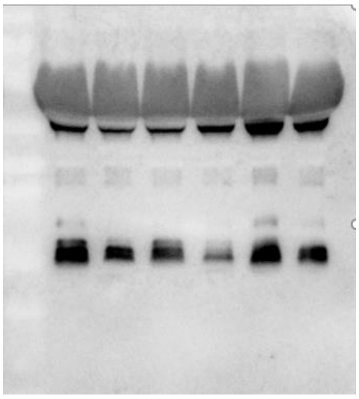

Middle: Apo A-I, others: non-specific bands; **Up black (Albumin)**

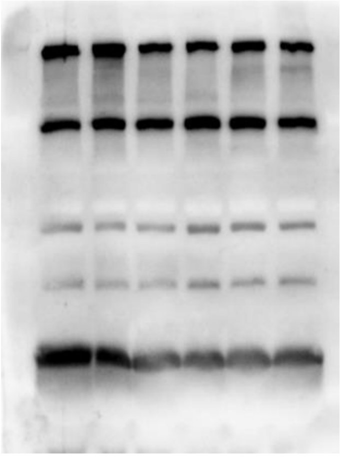

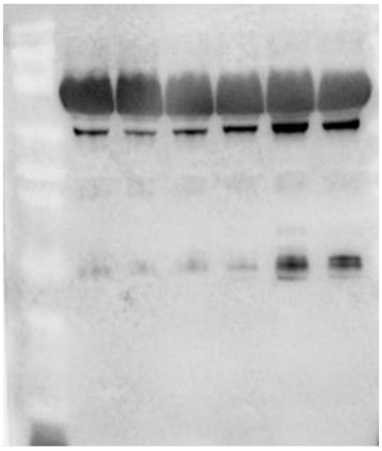

Up two (Apo B), down: non-specific bands; **Up black (Albumin)**

Liver H & E staining

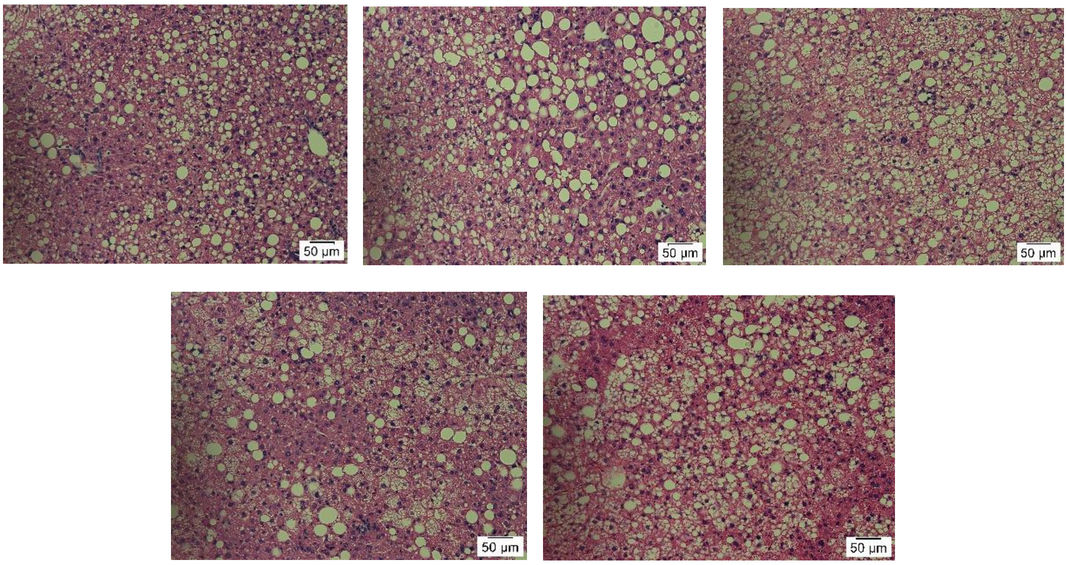

Vehicle

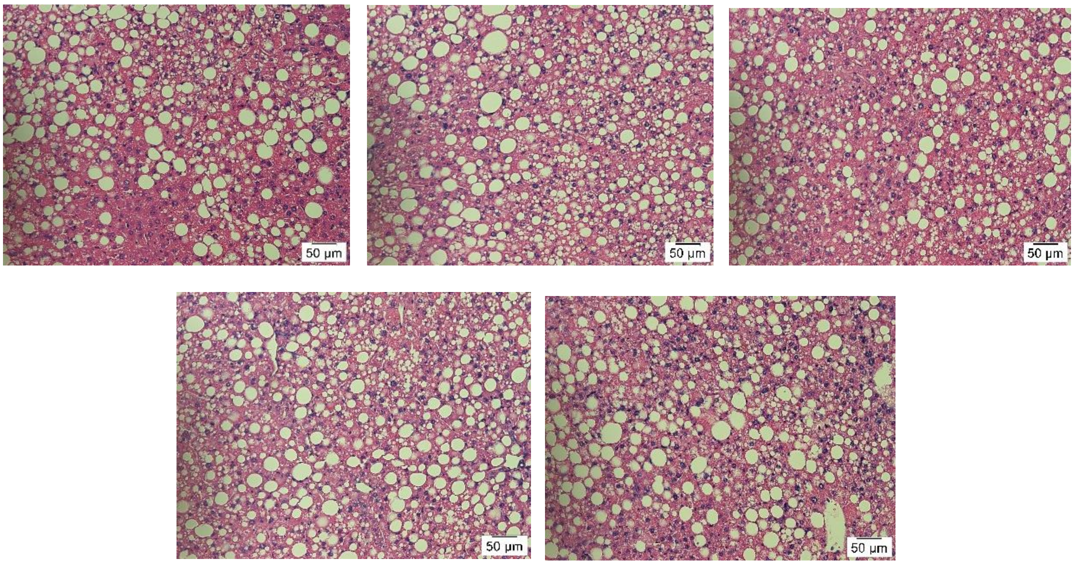

Fenofibrate

Western blotting (Liver)

β-actin

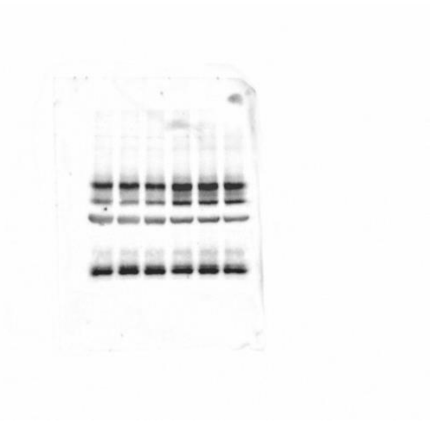

PPARα (Up)

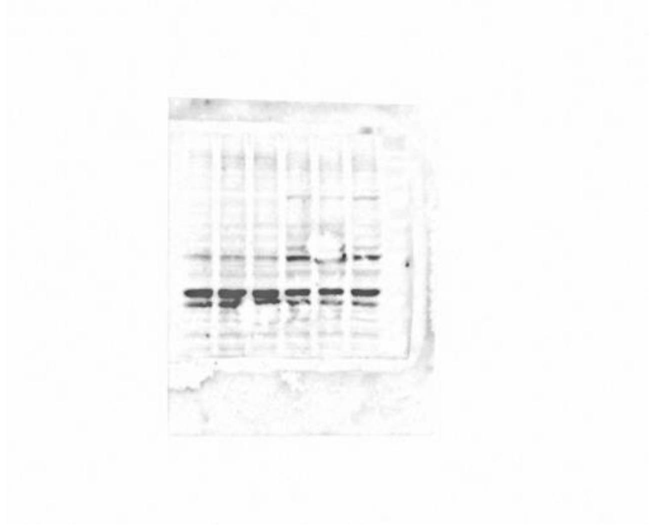

Up: PPARγ Down: β-actin

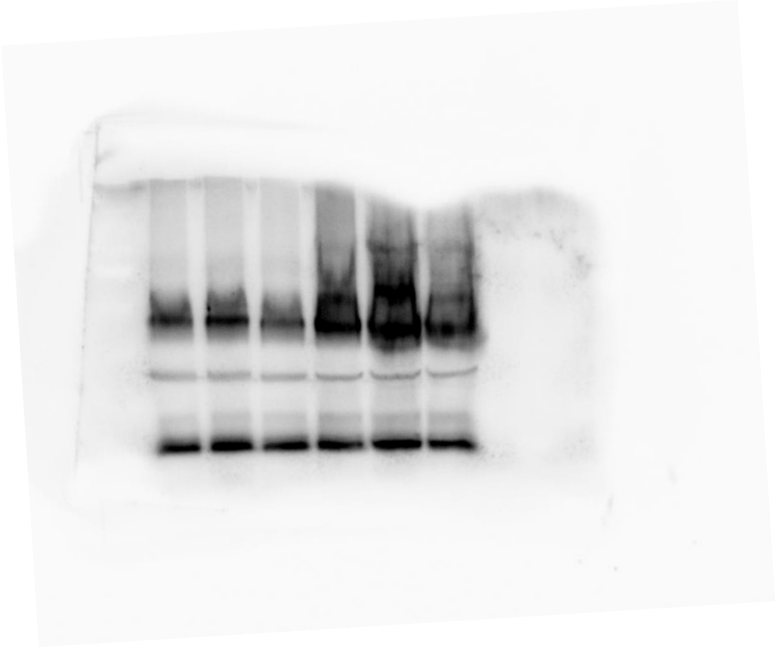

Up: SREBP-1c Down: β-actin

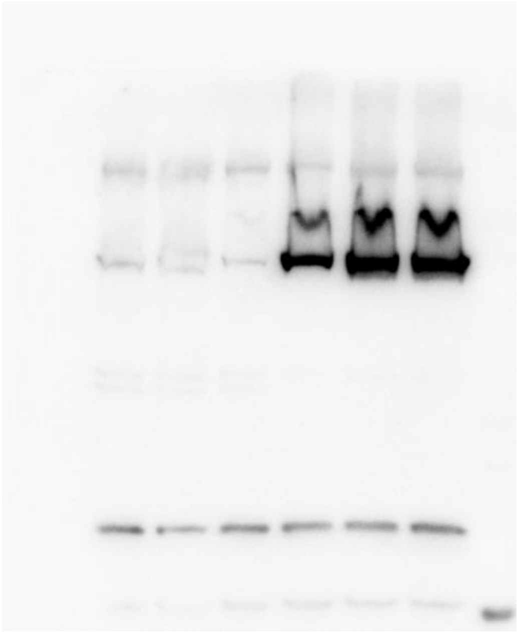

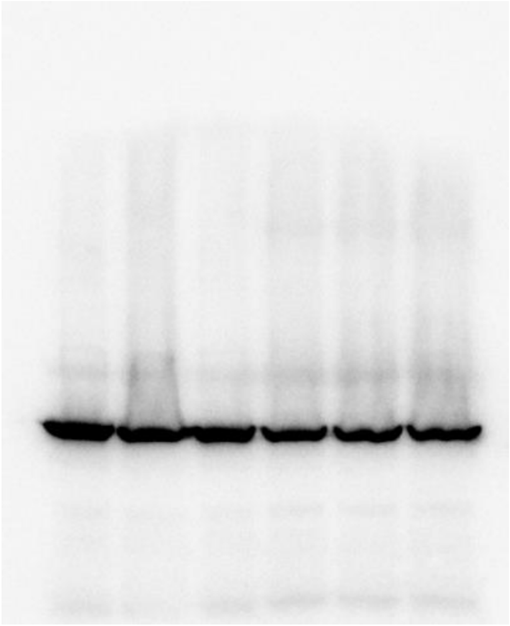

Up bands: LDLR β-actin

Western blotting (Fat tissue)

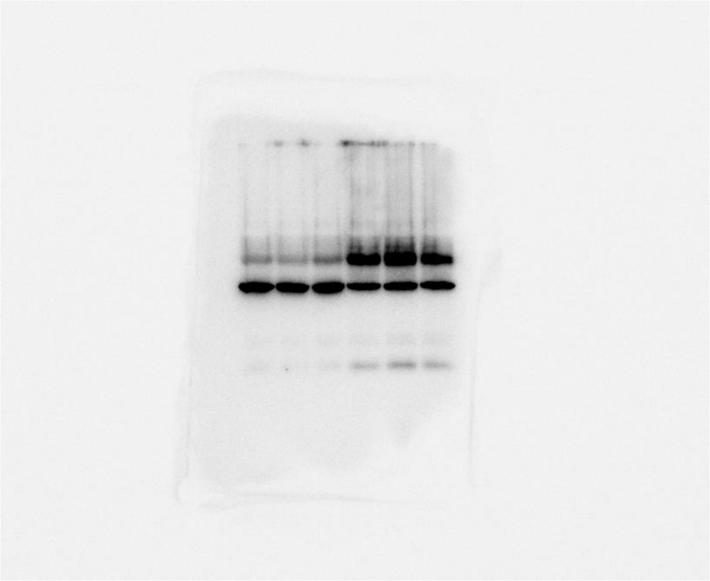

Up: PPARα Down: β-actin

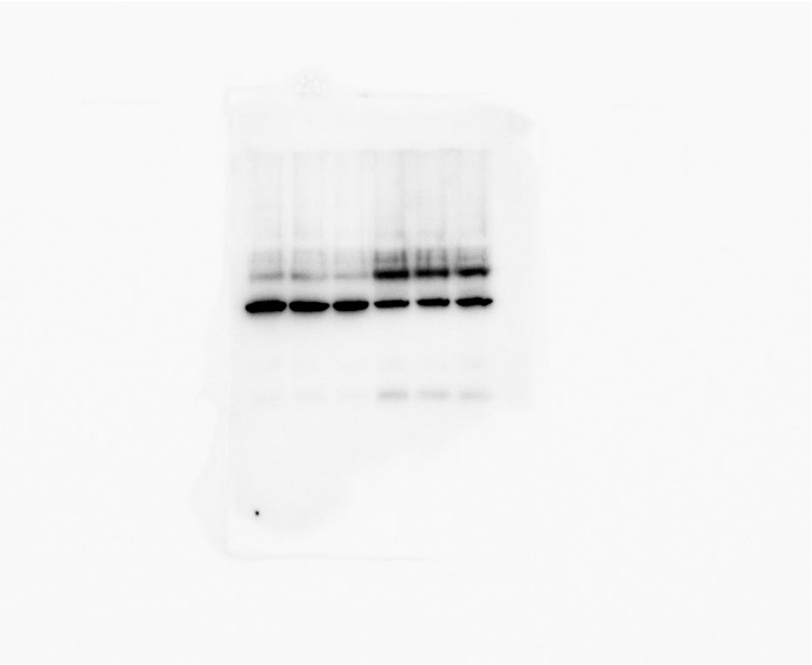

Up: PPARγ Down: β-actin

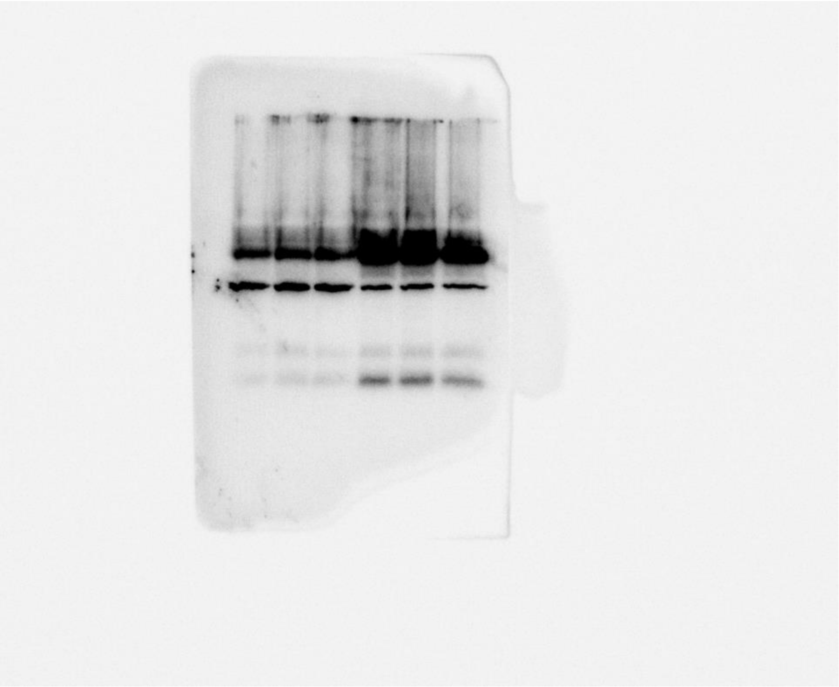

Up: srebp1c Down: β-actin
